# Collective Sensing of β-Cells Generates the Metabolic Code

**DOI:** 10.3389/fphys.2018.00031

**Published:** 2018-01-24

**Authors:** Dean Korošak, Marjan Slak Rupnik

**Affiliations:** ^1^Institute for Physiology, Faculty of Medicine, University of Maribor, Maribor, Slovenia; ^2^Faculty of Civil Engineering, Transportation Engineering and Architecture, University of Maribor, Maribor, Slovenia; ^3^Percipio Ltd., Maribor, Slovenia; ^4^Center for Physiology and Pharmacology, Institute for Physiology, Medical University of Vienna, Vienna, Austria; ^5^Alma Mater Europaea - European Center Maribor, Maribor, Slovenia

**Keywords:** collective sensing, pancreatic islets, spin glass models, metabolic code, Ca^2+^ imaging, Ca^2+^ signaling, correlations, intercellular communication

## Abstract

Major part of a pancreatic islet is composed of β-cells that secrete insulin, a key hormone regulating influx of nutrients into all cells in a vertebrate organism to support nutrition, housekeeping or energy storage. β-cells constantly communicate with each other using both direct, short-range interactions through gap junctions, and paracrine long-range signaling. However, how these cell interactions shape collective sensing and cell behavior in islets that leads to insulin release is unknown. When stimulated by specific ligands, primarily glucose, β-cells collectively respond with expression of a series of transient Ca^2+^ changes on several temporal scales. Here we reanalyze a set of Ca^2+^ spike trains recorded in acute rodent pancreatic tissue slice under physiological conditions. We found strongly correlated states of co-spiking cells coexisting with mostly weak pairwise correlations widespread across the islet. Furthermore, the collective Ca^2+^ spiking activity in islet shows on-off intermittency with scaling of spiking amplitudes, and stimulus dependent autoassociative memory features. We use a simple spin glass-like model for the functional network of a β-cell collective to describe these findings and argue that Ca^2+^ spike trains produced by collective sensing of β-cells constitute part of the islet metabolic code that regulates insulin release and limits the islet size.

## 1. Introduction

Endocrine cells in vertebrates act both as coders and decoders of metabolic code (Tomkins, 1975) that carries information from primary endocrine sensors to target tissues. In endocrine pancreas, energy-rich ligands provide a continuous input to a variety of specific receptor proteins on and in individual β-cells and initiate signaling events in and between these cells (Henquin, 2009). In an oversimplified medical physiology textbook interpretation, glucose is transported into a β-cell through facilitated diffusion, is phosphorylated and converted within a metabolic black box to ATP, leading to closure of K_*ATP*_ channels, cell membrane depolarization and activation of voltage-activated calcium channels (VACCs), followed by a rise in cytosolic Ca^2+^ to a micromolar range and triggering of SNARE-dependent insulin release (Ashcroft and Rorsman, 1989). However, glucose may influence β-cells signaling through several additional routes. There may be alternative glucose entry routes, like for example active Na-glucose cotransport (Tomita, 1976; Trautmann and Wollheim, 1987), alternative calcium release sites, like ryanodine (Islam, 2002) and IP_3_ receptors (Lang, 1999) or glucose may directly activate the sweet taste receptor and initiate signaling (Henquin, 2012), to name a few. Activation of a β-cell on a single cell level therefore likely involves triggering of a variety of elementary Ca^2+^ events (Berridge et al., 2000), which interfere in space and time into a unitary β-cell Ca^2+^ response to support Ca^2+^-dependent insulin release. This Ca^2+^-dependent insulin release can be further modulated by activation of different protein phosphorylation/dephosphorylation patterns (PKA, PKC, Cdk5, etc.) (Mandic et al., 2011; Skelin and Rupnik, 2011) or other protein modifications (Paulmann et al., 2009) to either reduce or increase the insulin output.

One of the important features of the sensory collectives is the optimization of the spatial relations between its elements to maximize the precision of sensing (Fancher and Mugler, 2017; Saakian, 2017). In islets of Langerhans, β-cells dwell as morphologically well defined cellulo-social collectives. These ovoid microorgans are typically not longer than 150 μm. The relatively small and constant pancreatic islet size is an intriguing feature in vertebrate biology. The size distribution of islets is comparable in humans, rodents and wider within different vertebrate species, irrespective of evident differences in overall body and pancreas size as well as total β-cell mass (Kim et al., 2009; Dolenšek et al., 2015). In mice, islet sizes range between 50 and 600 μm, with a median values below 150 μm (Lamprianou et al., 2011). To accommodate differences in the body size, there is nearly a linear relationship between the total number of similarly sized islets and body mass across different vertebrate species (Montanya et al., 2000; Bouwens and Rooman, 2005). However, why are islets so conserved in size is unknown.

All β-cells within an islet collective represent a single functional unit, electrically and chemically coupled network, with gap junction proteins, Connexins 36 (Cx36) (Bavamian et al., 2007), for short-range interactions and with paracrine signaling (Caicedo, 2013) for long-range interactions between cells. The unitary cell response in one β-cell influences the formation of similar responses in neighboring β-cells and contributes to coordination of a large number of β-cells (Cigliola et al., 2013; Stožer et al., 2013a). Explorations of these functional β-cell networks, constructed from thresholded pairwise correlations of Ca^2+^ imaging signals (Stožer et al., 2013b; Markovič et al., 2015; Johnston et al., 2016; Gosak et al., 2017a), showed that strongly correlated subsets of β-cell collective organize into modular, broad-scale networks with preferentially local correlations reaching up to 40 μm (Markovič et al., 2015), but understanding of mechanisms that lead to these strongly correlated networks states in β-cell populations is still lacking. We argue that β-cells sense, compute and respond to information as a collective, organized in a network similar to sensory neuron populations (Schneidman et al., 2006; Tkačik and Bialek, 2016), and not as a set of independent cells strongly coupled only when stimulation is high enough.

Here we reanalyze pairwise correlations of Ca^2+^ spike trains (unitary β-cell responses on the shortest temporal scale) in β-cell collective recorded in fresh pancreatic tissues slice under changing glucose stimulation conditions (6 mM subthreshold–8 mM stimulatory) using methodological approaches previously described (Stožer et al., 2013b; Markovič et al., 2015; Gosak et al., 2017a,b). We specifically look at weak correlations between β-cells which we found to be widely spread across the islet (Azhar and Bialek, 2010). Guided by the use of statistical physics models in describing populations of neurons (Schneidman et al., 2006; Tkacik et al., 2009), we use a simple spin glass model for Ca^2+^ β-cells activity and show that it well captures the features observed in the measured data. In a way, we recognize this efficiency of simple models in both neuronal and endocrine cell collectives as one manifestation of the “beauty in function” (Rasmussen, 1970).

## 2. Spin model of a β-cell collective

Spin models have been borrowed from statistical physics to describe the functional behavior of large, highly interconnected systems like sensory neurons (Schneidman et al., 2006; Tkacik et al., 2009; Tkačik et al., 2014), immune system (Parisi, 1990), protein interactions (Bryngelson and Wolynes, 1987), financial markets (Bornholdt, 2001; Krawiecki et al., 2002), and social interactions between mammals (Daniels et al., 2016, 2017).

The model of the islet consist of *N* cells; at time *t* each of the cells can be in one of two states, spiking or silent, represented by a spin variable *S*_*i*_(*t*) = ±1, (*i* = 1, …, *N*). The discrete time steps in model computations correspond to 2 s binning size of the Ca^2+^ data. The effective field *E*_*i*_ of the i-th cell has two contributions: one from the cell interacting with all other cells with interaction strength *J*_*ij*_, and one from external field *h*_*i*_. We assume that interactions extend over the whole system.

(1) $$\begin{array}{r} E_{i}\left(t\right)=h_{i}\left(t\right)+\overset{N}{\underset{j=1}{\sum }}J_{ij}S_{j}\left(t\right) \end{array}$$

At the next moment (t + 1) each cell updates its state *S*_*i*_(*t*) with the probability *p* to *S*_*i*_(*t* + 1) = +1 and with the probability 1 − *p* to *S*_*i*_(*t* + 1) = −1. The probability *p* depends on the effective field *E*_*i*_ that the i-th cell senses:

(2) $$\begin{array}{r} p=\frac{1}{1+\exp\left(-2E_{i}\right)}. \end{array}$$

The interaction strength *J*_*ij*_ is a fluctuating quantity with contributions from amplitude *J* common to all links and from the pairwise contributions with amplitude I (Krawiecki et al., 2002): *J*_*ij*_ = *Jλ*(*t*)+*Iν*_*ij*_(*t*). Here are the fluctuations λ(*t*) and ν_*ij*_(*t*) random variables uniformly distributed in the interval [−1, 1]. The external field *h*_*i*_(*t*) = η(*t*) is also a random variable, uniformly distributed in the interval η(*t*) = [*h*_*min*_, *h*_*max*_]. In the mean-field approximation the average state of the system $m\left(t\right)=\frac{1}{N}\underset{j}{\sum }S_{j}$, evolves with time according to Krawiecki et al. (2002):

(3) $$\begin{array}{r} m\left(t+1\right)=\tanh\left(J\lambda \left(t\right)m\left(t\right)+h_{mf}\left(t\right)\right), \end{array}$$

where we *h*_*mf*_ = η(*t*)/*N*. In the Results section below we demonstrate that the model describes the important features observed in the data well. In all computations we used a model with *N* = 200 spins, and we set, following the original model (Krawiecki et al., 2002), the pairwise interaction amplitude to *I* = 2*J*. The values of the remaining three free parameters of the model, *J*, *h*_*max*_ and *h*_*min*_, were chosen to fit the model computations to the qualitative features of the Ca^2+^ data as described in the next section.

## 3. Results

The functional multicellular imaging (fMCI) records a full temporal activity trace for every cell in an optical plane of an islet from which meaningful quantitative statements about the dynamics of unitary Ca^2+^ responses and information flow in the β-cell collective are possible (Dolenšek et al., 2013; Stožer et al., 2013a). Briefly, after the stimulation with increased glucose level, first asynchronous Ca^2+^ transients appear, followed by a sustained plateau phase with oscillations on different temporal scales, from slow oscillations (100–200 s) to trains of fastest Ca^2+^ spikes (1–2 s). As the relation between the rate of insulin release and cytosolic Ca^2+^ activity shows saturation kinetics with high cooperativity (Skelin and Rupnik, 2011), the insulin release probability is significantly increased during these Ca^2+^ spikes.

Initially, fMCI has been done at the glucose concentrations much higher than those at which β-cells usually operate. The main reason for this was to ensure comparability of the results with the mainstream research in the field using mostly biochemical approaches. At 16 mM glucose, a collective of β-cells responds in a fast, synchronized, and step-like manner. Therefore the first interpretation has been that gap junction coupling between neighboring β-cells presents a major driving force for the β-cell activation and inhibitory dynamics (Hraha et al., 2014; Markovič et al., 2015). Accordingly, the removal of Cx36 proteins does cause hyperinsulinemia at resting glucose levels and blunted responses to stimulatory glucose concentration (Speier et al., 2007). Since β-cells in fresh pancreatic tissue slices are sensitive to physiological concentration of glucose (6–9 mM) (Speier and Rupnik, 2003), we here focused on this less explored concentration range. We looked at the spiking part of the Ca^2+^ imaging signals for which it has been previously shown to contain enough information to allow reconstruction of functional cell networks (Stetter et al., 2012).

**Figure 1:**
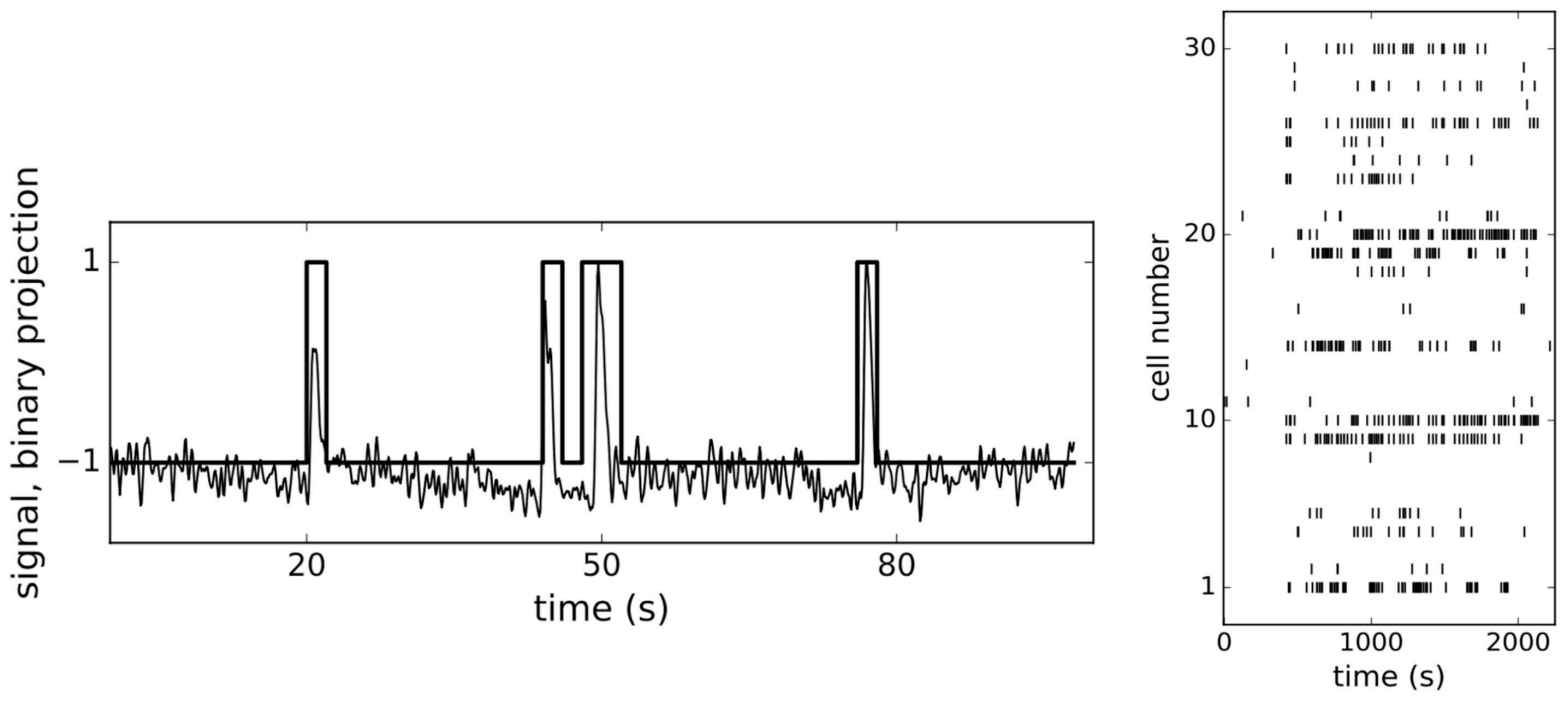
**(Left)** A Ca^2+^ trace showing a short train of spikes after ensemble empirical mode decomposition with overlaid binary form with 2 s wide bins. **(Right)** Spin raster plot of 30 randomly picked β-cells.

For the present analysis we reused a dataset of individual Ca^2+^-dependent events from *N* = 188 ROIs with known positions from the central part of the fresh rodent pancreatic oval shaped islet (370 um in length and 200 um wide), representing β-cells, recorded with fMCI technique at 10 Hz over period of 40 min (for methodological details see Stožer et al., 2013b; Markovič et al., 2015; Gosak et al., 2017a,b). During the recording the glucose concentration in the solution filling the recording chamber has been increased from 6–8 mM, reaching equilibration at around 200 s after the start of the experiment, and then decreased to initial concentration near the end of experiment at around 2,000 s (dashed red lines in **Figures 4**, **5** represent points where glucose levels were completely equilibrated in the recording chamber). We applied ensemble empirical mode decomposition (Luukko et al., 2016) on recorded traces to isolate the Ca^2+^ spiking component of the signal. Finally, based on previous experiments in our laboratory, we binarized the signals using 2 s wide bins (Figure 1, left panel) and obtained binary spike trains *S*_*j*_(*t*)±1, (*j* = 1…*N*), of β-cells' Ca^2+^ activity, each cell represented as a spin. As can be seen from the Figure 1 the chosen bin width adequately describe the unitary events seen in the calcium traces. An example of spiking dynamics of 30 randomly chosen spins is shown as a raster plot in the right panel of Figure 1.

**Figure 2:**
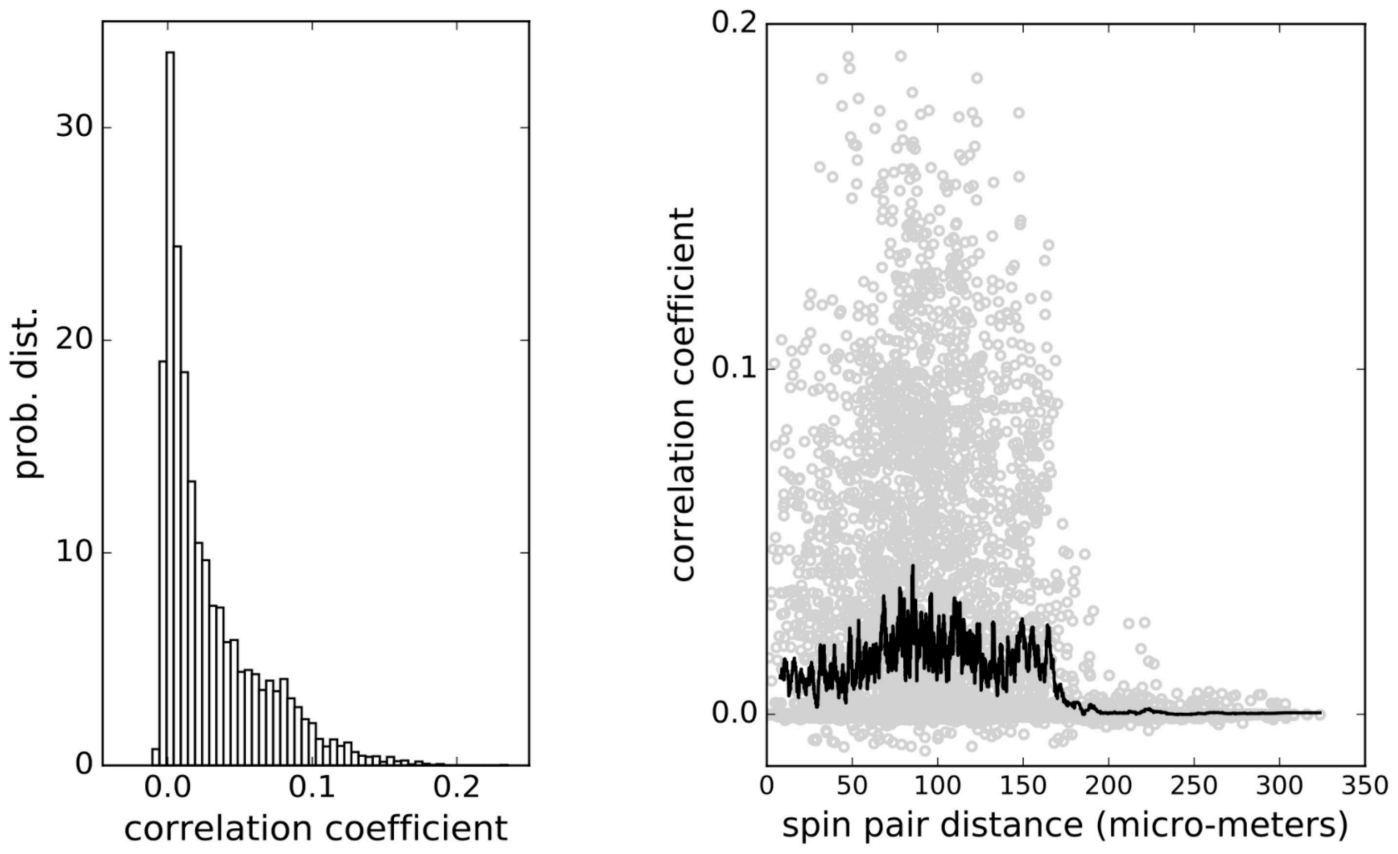
**(Left)** Distribution of pairwise correlations of β-cell collective computed from Ca^2+^ imaging spiking signals. **(Right)** Pair correlations distribution over distance. Weak correlations extend over the whole system up to 170 μm. Black line shows the average values of correlations at particular cell-cell distances.

Statistical methods based on mostly pairwise correlations between neurons populations have been successfully used in predicting spiking patterns in cell populations (Schneidman et al., 2006; Tkacik et al., 2009; Tkačik et al., 2014; Ferrari et al., 2017). It may seem surprising that models with first and second-order correlation structure work not only when the cell activity is very sparse so the correlations could be described by perturbation theory (Roudi et al., 2009), but can reproduce the statistics of multiple co-spiking activity (Barton and Cocco, 2013; Merchan and Nemenman, 2016; Ferrari et al., 2017). We computed truncated correlations

(4) $$\begin{array}{r} c\left(i,j\right)=\langle S_{i}S_{j}\rangle -\langle S_{i}\rangle \langle S_{j}\rangle \end{array}$$

for all pairs of cells. The pairwise correlations found are mostly weak with the distribution shown in Figure 2 (left panel), but they extend widely over the distances up to 170 μm across the islet, which is larger than an average vertebrate islet size (Figure 2, right panel). At distances larger than 170 μm the correlations decrease sharply toward zero. Such weak and long-ranging pairwise correlations could be the root of criticality and of strongly correlated network states in biological systems (Schneidman et al., 2006; Azhar and Bialek, 2010; Mora and Bialek, 2011; Tkačik et al., 2015).

**Figure 3:**
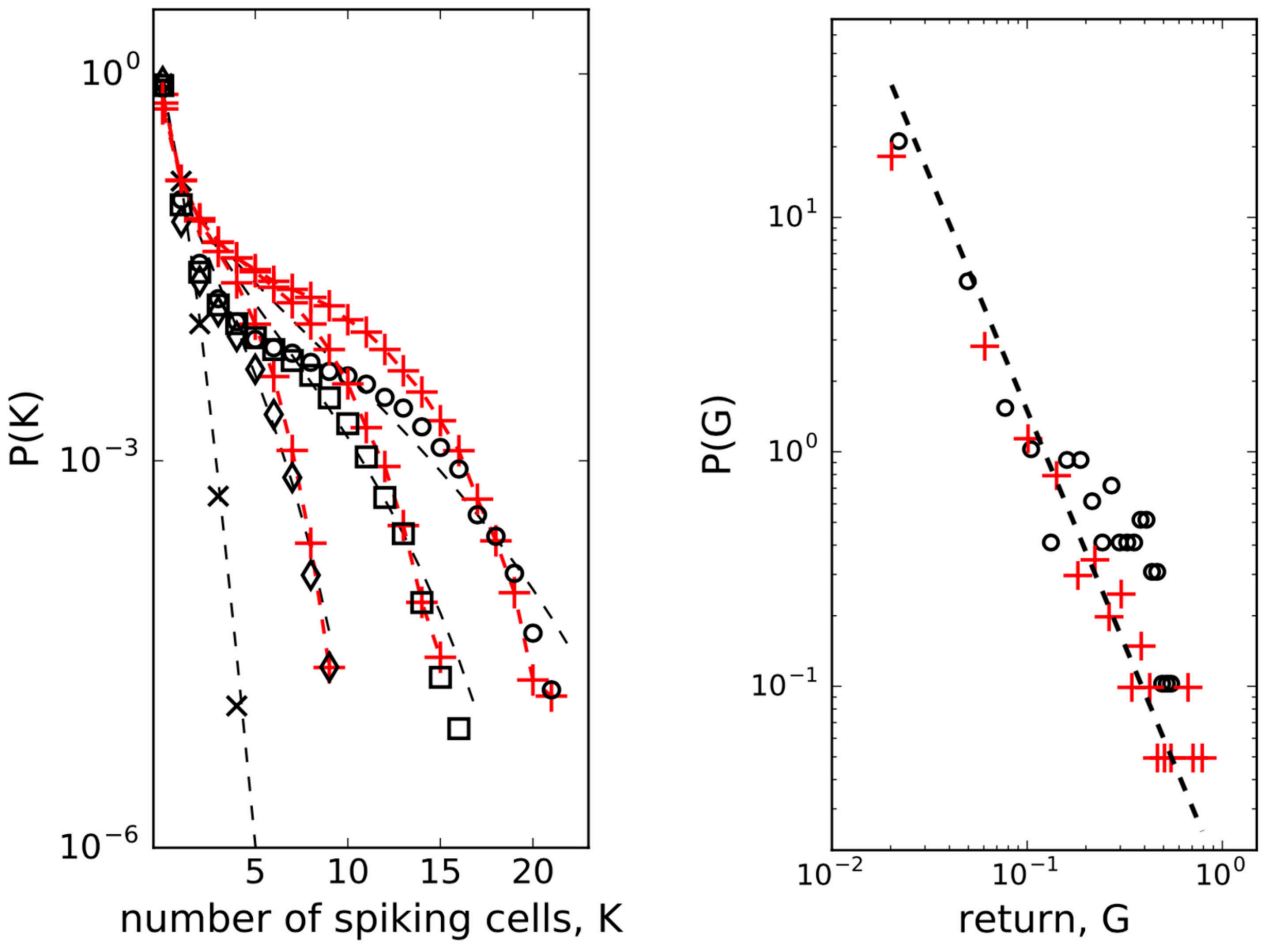
**(Left)** Probability distributions of *K* cells among *N* spiking simultaneously. Randomly shuffled spike trains (black crosses, *N* = 10) with dashed line - Poisson distribution; *N* = 10 (diamonds), *N* = 20 (squares), *N* = 30 (open dots); model (red pluses + red dashed line with *J* = 2.0 used for the entire dataset, *N*_*spins*_ = 200 spins, *h*_*min*_ = −2.65, *h*_*max*_ = −1.65), beta-binomial model (Nonnenmacher et al., 2017) (black dashed line, α = 0.38, β = 11.0); **(Right)** Scaling of mean field return: open dots - data, red pluses - mean field approximation from the spin model of β-cells computed with *J* = 2.0, *h*_*mf*_ = η(*t*)/*N*. Dashed line *P*(*G*) ~ *G*^−2.0^

To check for the existence of strongly correlated states in weakly correlated β-cell collective we computed probability distributions *P*_*N*_(*K*) of *K* simultaneously spiking cells in groups of *N* = 10, 20, 30 cells. Here, we used the entire dataset, the low and the high glucose concentration parts, from which we sampled cells signals. While the *P*_*N*_(*K*) of randomly reshuffled spike trains expectedly follows Poisson distribution (left panel in Figure 3, black crosses and dashed line for *N* = 10 spins), the observed co-spiking probabilities are orders of magnitude higher (diamonds in left panel of Figure 3 for *N* = 10 spins) than corresponding probabilities in groups of independent spins. The statistics of these co-spiking events were described by an exponential distribution (Schneidman et al., 2006), by finding the effective potential (Tkačik et al., 2013, 2014) matching the observed *P*_*N*_(*K*) and adding it to the hamiltonian of the model, or by using beta-binomial distribution (Nonnenmacher et al., 2017) *P*_*N*_(*K*) = *C*(*N, K*)*B*(α + *K*, β + *N* − *K*)/*B*(α, β) where *C*(*N, K*) is binomial coefficient and *B*(α, β) is the beta function.

We next run the spin model of 200 β-cells and then sampled the computed spike trains to obtain *P*_*N*_(*K*) from the model for *N* = 10, 20, 30. Despite its simple structure, the model matches order of magnitude of the observed *P*_*N*_(*K*) well when we set the interaction strength at *J* = 2.0, as shown in the left panel of Figure 3 (red pluses and red dashed line), particularly for larger *K* values. In the model here we did not treat the low and the high glucose concentration part separately, we used *J* = 2.0 for the entire dataset. For comparison, we also show how the beta-binomial model fits to the observed data using the parameters α = 0.38, β = 11.0 in all *N* = 10, 20, 30 cases. These values are also close to the best-fitting parameters (α = 0.38, β = 12.35) to the simulated and observer correlated neural population activity data as reported in Nonnenmacher et al. (2017).

The microscopic model of interacting spins with interactions randomly varying in time (Krawiecki et al., 2002), adopted here to describe interacting β-cell collective, exhibits scaling of price fluctuations (Bornholdt, 2001) observed in financial markets (Gopikrishnan et al., 1999) and on-off intermittency with attractor bubbling dynamics of average price (Krawiecki et al., 2002). Following this idea, we looked at the logarithmic return of average state of β-cell collective at time *t* (Bornholdt, 2001): *G*(*t*) = log(*m*(*t*)) − log(*m*(*t* − 1)). As presented in the right panel of Figure 3, the distribution *P*(*G*) (of positive *G* values) can indeed be approximated with a scaling law: *P*(*G*) ~ *G*^−γ^ with γ = 2.0. There is an analytical relationship (Krawiecki et al., 2002) between *J* and exponent γ of the distribution of amplitudes of the return of the mean field, *J* = γ^1/(γ−1)^, which gives *J* = 2.0 for γ = 2.0. We used this as a consistency check between the model computations and mean field approximation. Computing the average state with the Equation (3) of the model, we can reproduce the observed distribution by setting on the interaction strength to *J* = 2.0 at *t*_*on*_ = 400 s and off to *J* = 0 at *t*_*off*_ = 2,200 s. The amplitude of the interaction *J* is consistent with the computation of the co-spiking probability.

**Figure 4:**
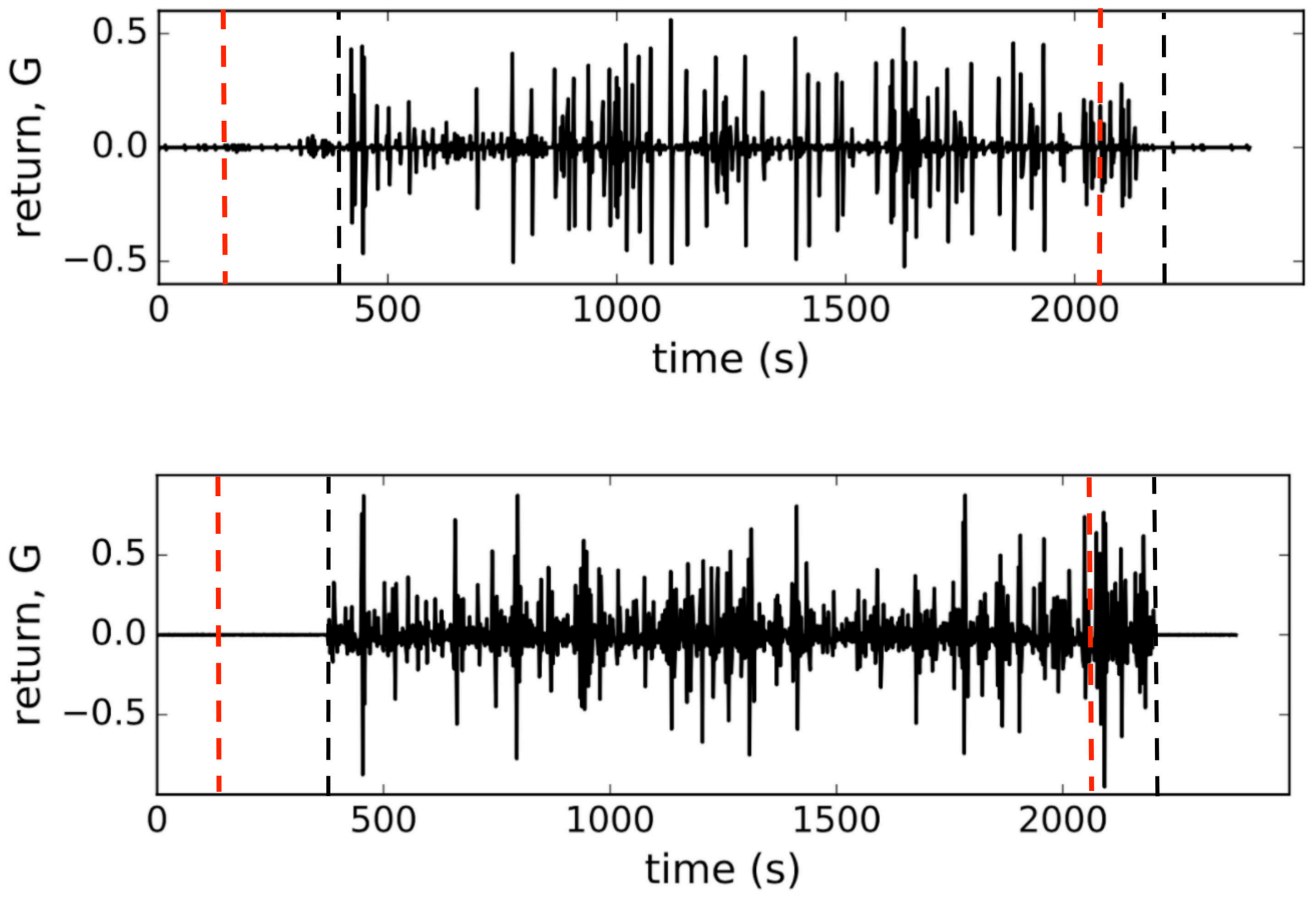
**(Upper)** Observed logarithmic return of the average state of of β-cell collective *G*(*t*), **(Lower)** logarithmic return of the average state computed from the model with *J* = 2.0 for *t*_*on*_ < *t* < *t*_*off*_, denoted with vertical dashed lines in figures. Dashed red lines represent points where glucose levels were completely equilibrated in the recording chamber.

**Figure 5:**
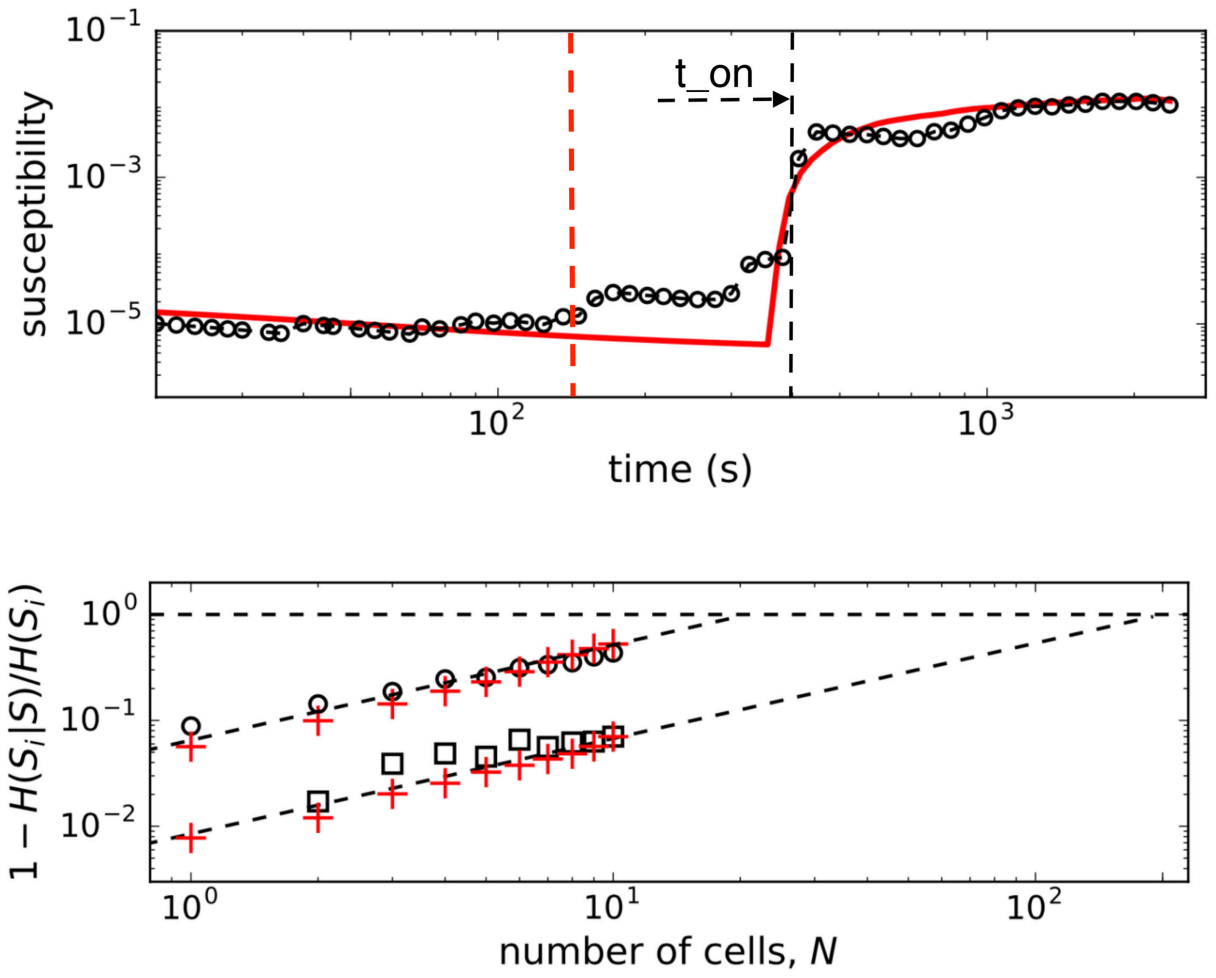
**(Upper)** Susceptibility of β-cell collective around transition to stimulatory glucose level. Open dots are the experimental data, red line shows the result of the mean field computations with *J* = 2.0 onset at *t* = *t*_*on*_(*blackdashedline*). Dashed red line represent the point where glucose level completely equilibrated during the 6–8 mM transition in the recording chamber. **(Lower)** Normalized conditional entropy. Open dots are experimental data at 8 mM glc, open squares at 6 mM glc. Red pluses show the results of the spin model computations with *N*_*spins*_ = 200 spins, and the parameters: *h*_*min*_ = −2.65, *h*_*max*_ = −1.65, *J* = 2.0 for the upper, and *J* = 0 for lower the lower part.

In Figure 4 we show the plots of both, observed and computed, returns of average state of interacting β-cells for comparison. The glucose concentration was changed during the experiment in a stepwise manner: from 6 to 8 mM at the beginning and back to 6 mM near the end of recording period. The effect of both changes is nicely visible in the *G*(*t*) plot (upper panel, Figure 4) where the on-off intermittent dynamics of the average state starts around *t*_*on*_ = 400 s and lasts until around *t*_*off*_ = 2, 200 s in the experiment. Both observed events are delayed with respect to the times of glucose concentration change due to the asynchronous Ca^2+^ transients (Stožer et al., 2013a). We expect that the response of β-cell collective to the stimulus increase must be visible in the variance of average state Var(*m*) which is in Ising-like model we are using here equal to susceptibility of the system χ = Var(*m*) = 〈*m*^2^〉−〈*m*〉^2^. We used the low glucose concentration part (6 mM) of the data to estimate the boundaries of the external field interval [*h*_*max*_, *h*_*min*_] to describe the first part of susceptibility. Using the maximal and minimal spiking rates of cells (*m*_*max*_, *m*_*min*_) in 6 mM glucose from the data and the mean-field approximation with *J* = 0 corresponding to the non-stimulatory glucose regime we have $\left[h_{max},h_{min}\right]=\left[\tanh^{-1}\left(m_{max}\right),\tanh^{-1}\left(m_{min}\right)\right]=\left[-1.65,-2.65\right]$. In upper panel of Figure 5 (open black dots) we show the plot of susceptibility as a function of recording time, focusing around the transition to increased glucose concentration during the experiment. There is a sharp increase of susceptibility at around *t*_*on*_, the same time the on-off intermittency starts to appear in *G*(*t*). Using mean field approximation of the spin model Equation (3) for computation of susceptibility (averaged over many runs) and setting *J* = 0 for *t*<*t*_*on*_ and *J* = 2.0 for *t*>*t*_*on*_ we can well describe the observed evolution of susceptibility and capture the rapid onset of increased sensibility of the islet (red line in upper part of Figure 5).

Pairwise correlation structure enables error-correction features of population coding in neural systems (Schneidman et al., 2006). To check for memory-like or error-correcting properties in islets, we use the conditional entropy *H*(*S*_*i*_|*S*), the measure for the information we need to determine the state of *N*-th cell (i.e., spiking or not) if we know the states of *N* − 1 cells (*S* = *S*_*j*_ ≠ *i*) in a group of *N* cells. If the state of the *N*-th cell is completely determined by other *N* − 1 cells, the conditional entropy is zero *H*(*S*_*i*_|*S*) = 0 and the error correction is perfect. When *S*_*j*_ are independent random states, the conditional entropy equals the entropy of the *N*-th cell *H*(*S*_*i*_).

We computed the quantity 1 − *H*(*S*_*i*_|*S*)/*H*(*S*_*i*_) (normalized mutual information) as a function of number of cells (for small groups of cells) and extrapolate the trend toward the limit *H*(*S*_*i*_|*S*) = 0 that determines the critical number of cells, *N*_*c*_, needed to predict the state of another cell. As seen in the lower panel of Figure 5, the predictability is a glucose-dependent parameter. With non-stimulatory glucose concentration, the complete set of data is required for predictions, whereas at 8 mM glucose we find that order of magnitude smaller number of measured cells are needed to predict the states of other cells.

## 4. Discussion

Pancreatic β-cell continuously intercepts a variety of energy-rich or signaling ligands using the whole spectrum of specific receptors on the cell membrane, as well as in metabolic and signaling pathways within the cell. The cell converts these signals into a binary cellular code, for example a train of Ca^2+^ spikes, which drive insulin release that fits current physiological needs of the body. This allow already a single cell to sense its chemical environment with extraordinary, often diffusion limited precision (Bialek and Setayeshgar, 2005), however, judging by their heterogeneous secretory behavior in cell culture, the precision of sensing among the individual β-cells is quite diverse (Hiriart and Ramirez-Medeles, 1991). Recent experimental evidence and modeling have shown that cell collectives sense better compared to an individual cell. The precise mechanism of this collective sensing improvement depends on cell-cell communication type, which can be short-range with direct cell contacts or long-range with paracrine signaling (Fancher and Mugler, 2017; Saakian, 2017). Furthermore, also long-range interaction have its finite reach which can poise a limit to the cell collective size and therefore determines its optimal as well as maximal size. As mentioned in the Introduction here, it is intriguing how well conserved the pancreatic islet size is in vertebrates of dramatically different body dimensions (Montanya et al., 2000). In a single vertebrate organism the size of the islets can be bigger that 150 um, but functional studies revealed that the islets bigger than 200 um secrete 50% less insulin after glucose stimulation (Fujita et al., 2011). These functional differences between small and large islets have been partially attributed to diffusion barriers for oxygenation and nutrition, limiting the survival of core β-cells in bigger islets after isolation. However, reducing these diffusion barriers had no influence on insulin secretory capacity (Williams et al., 2010) suggesting that other factors, like diffusion of paracrine signaling molecules (Caicedo, 2013), could limit the collective β-cell function in bigger islets. This dominance of a long-range information flow, likely limited to some physical constraints, has indicated the use of the mathematical equivalency with spin glass-like systems (Tkačik and Bialek, 2016).

We strongly believe that advanced complex network analysis based on strong short-range correlations can continue to provide valuable information regarding the β-cell network topologies, network on network interactions and describe the functional heterogeneity of individual β-cells (Gosak et al., 2015, 2017a; Markovič et al., 2015; Johnston et al., 2016). However, the main goal of the present study was to determine the influence of weak long-range correlations between pairs of β-cells on the probability of activation of single β-cells. Recently has been shown that it suffice to use pairwise correlations to quantitatively describe the collective behavior of cell collectives (Merchan and Nemenman, 2016). The typically small values of pairwise correlation coefficients with the median values below 0.02, would intuitively be ignored and β-cells described as if they act independently, however in larger populations of cells this assumption completely fails (Schneidman et al., 2006). In fact, at physiological stimulatory glucose levels between 6 and 9 mM, β-cell collectives are entirely dominated by weak average pairwise correlations (Figure 2). Nevertheless, this is the glucose concentration range, where β-cells are most responsive to the nutrient to, as a collective, compute their activity state and pulsatile insulin release, and to meet the organismal needs between the environmental and behavioral extremes of food shortage and excess (Schmitz et al., 2008)?

Based on the range of the calculated weak pairwise correlations of up to 170 um (Figure 2), we predict that β-cells collective falls into a category of sparse packed tissues with dominant paracrine interactions and that cell-cell distances contribute to optimal sensing and functional response in creating the metabolic code governing the release of insulin. It remains unclear whether and how the position of β-cells within an islet is controllable. As many other cells, β-cells are polarized and possess a primary cilium (Gan et al., 2017), which should have a primary role in sensory function, i.e., insulin sensing in paracrine signaling (Doğaner et al., 2016), and not in cell motility. It is quite interesting though, that the ciliopathies are highly associated with reduced β-cell function and increased susceptibility to diabetes mellitus (Gerdes et al., 2014). Future experiments are required to test for the possible motility of β-cells within the islet to adopt an optimal separation of key sensitive β-cells. To further extrapolate the collective sensing idea, it is also possible that the diffuse arrangement of a collective of islets within different parts of pancreas, which are exposed to different vascular inputs (Dolenšek et al., 2015), serves to optimize nutrient sensing experience, yet on a higher organizational level, providing a topological information regarding the nutrient levels in different parts of the gastrointestinal tract. The nature and level of interactions between individual islets in the pancreas are currently also unknown.

As in retinal neuron networks, β-cells encode information about the presence of energy-rich nutrients into sequences of intermittent Ca^2+^ spikes. In a natural setting of sensory neural networks with stimuli derived from a space with very high dimensionality the coding seems challenging and interpretations require some strong assumptions (Tkačik et al., 2014). We currently do not understand the input dimensionality of a typical ligand mixture around the β-cells, we simply assume it is not high. As in retinal networks (Schneidman et al., 2006; Tkačik et al., 2014), the predictability regarding the functional state of individual β-cells is defined by the network and not the chemical environment. This suggests that the sensory information at physiological glucose levels is substantially redundant. It is likely that the nutrient mixture presents a noisy challenge for the information transfer which is typical for biological system. But why study the insulin release pattern or the metabolic code? The β-cell network possess associative or error-correcting properties (Figure 5), so this idea from the sensory neuron networks can be generalized also to populations of endocrine cells (Schneidman et al., 2006), which may again influence the optimal islet size and suggest the presence of functional subunits within the islet that could adapt, for example, to changing environment in a dynamic fashion. Furthermore, error-correction properties are glucose dependent and can be physiologically modulated (Figure 5). The trains of Ca^2+^ spikes at constant glucose stimulation (8 mM) are inhomogeneous, display on-off intermittency (Figure 4) and scaling of log returns of average state (Figure 3) analog to models of financial time series (Krawiecki et al., 2002). For the spin glass approach we also postulate that the sources of stochasticity in an islet collective are various. On one hand, the β-cells make decisions on activation under the influence of the external environment and other β-cells. Second, also the time-dependent interaction strength among β-cells is random, which could reflect their socio-cellular communication network and indicate that the external environment can be sensed differently between different β-cells in an islet (Gosak et al., 2017b).

Biological systems seem to poise themselves at criticality, with a major advantage of enhanced reactivity to external perturbations (Mora and Bialek, 2011). Often a limited number of individual functional entities, cell or groups of cells as found in pancreatic islets, appeared to be limiting to address criticality. However, it has been recently demonstrated that even in biological systems with small number of interacting entities one can operationally define criticality and observe changes in robustness and sensitivity of adaptive collective behavior (Daniels et al., 2017). Our results suggest that β-cells collective within the islet sits near its critical point and we could determine the susceptibility in the islet. Stimulatory glucose concentration (8 mM) has been decreasing distance to criticality by increasing sensitivity (Figure 5). Smaller distance to criticality at unphysiologically high glucose levels has its possible adverse consequences in a phenomenon called critical slowing down as the system takes more and more time to relax as it comes nearer to the critical point (Mora and Bialek, 2011). Our preliminary results show that at very strong stimulation (i.e., glucose levels above 12 mM) the whole system freezes into a certain state where short-term interaction take over enabling global phenomena within the islets, e.g., Ca^2+^ waves (Stožer et al., 2013b) requiring progressively longer periods to relax to baseline with increasing glucose concentrations.

Further work will be needed to exploit at what circumstances deviations in islet size can contribute to islet malfunction and pathogeneses of different forms of diabetes mellitus. Until recently it has been thought that insulin release is no longer functional in type 1 diabetes mellitus. We now know that even in type 1 diabetic patients small and functional collectives of β-cells persist in the pancreata of these patients even decades after the diagnosis (Faustman, 2014). On the other hand, the β-cells mass in an islet can be increased in type 2 diabetic patients in the initial phases after the diagnosis (Rahier et al., 2008) or in animal models (Daraio et al., 2017) and can only be reduced in the later phases (Rahier et al., 2008). The detailed relations between the reduced or increased insulin release, changed islet size and therefore changed circumstances for paracrine signaling in disturbed collective nutrient sensing and during the aforementioned pathogeneses of diabetes mellitus remain to be established.

## Author contributions

All authors listed, have made substantial, direct and intellectual contribution to the work, and approved it for publication.

### Conflict of interest statement

The authors declare that the research was conducted in the absence of any commercial or financial relationships that could be construed as a potential conflict of interest.
